# ICTV Virus Taxonomy Profile: *Guttaviridae*

**DOI:** 10.1099/jgv.0.001027

**Published:** 2018-02-08

**Authors:** David Prangishvili, Tomohiro Mochizuki, Mart Krupovic, ICTV Report Consortium

**Affiliations:** ^1^​ Department of Microbiology, Institut Pasteur, 25 rue du Dr. Roux, 75015 Paris, France; ^2^​ Earth-Life Science Institute, Tokyo Institute of Technology, Tokyo 152-8550, Japan

**Keywords:** *Guttaviridae*, ICTV report, taxonomy, Aeropyrum pernix ovoid virus 1

## Abstract

*Guttaviridae* is a family of enveloped viruses infecting hyperthermophilic archaea. The virions are ovoid or droplet-shaped, with a diameter of 55–80 nm and a length of 75–130 nm. The genome is a circular dsDNA molecule of around 14–20 kbp. The droplet-shaped morphology is unprecedented among viruses of bacteria and eukaryotes and represents a group of archaea-specific virion morphotypes. The family includes two genera, *Alphaguttavirus* and *Betaguttaviru*s, each with a single species. This is a summary of the International Committee on Taxonomy of Viruses (ICTV) Report on the taxonomy of *Guttaviridae,* which is available at www.ictv.global/report/guttaviridae.

## Virions

**Fig. 1.**
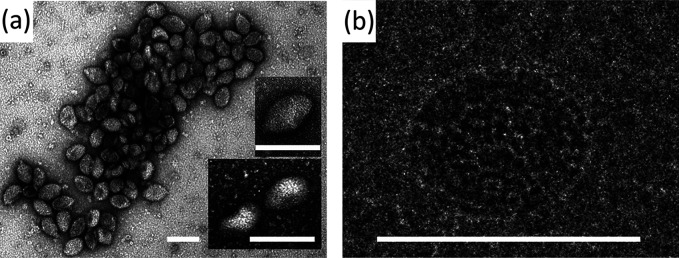
Electron micrographs of virions of Aeropyrum pernix ovoid virus 1. Virions were negatively stained (a) or embedded in ice (b). Scale bars, 100 nm. (Reproduced with permission from [2]. Copyright © 2011, American Society for Microbiology.)

Virions of Sulfolobus newzealandicus droplet-shaped virus (SNDV), the prototypical member of the genus *Alphaguttavirus*, are approximately 80×130 nm in size and display multiple fibres at the pointed end of the virion [1]. SNDV virions contain a major capsid protein of 17.5 kDa and at least two minor capsid proteins of 13.5 and 13 kDa, respectively [1]. Virions of Aeropyrum pernix ovoid virus 1 (APOV1), the sole representative of the genus *Betaguttavirus*, appear ovoid in cryo-electron micrographs, with dimensions of 55×75 nm (Table 1, Fig. 1) [2], i.e. 1.5 times smaller than those of SNDV. In negative-contrast electron micrographs, APOV1 virions are slightly pleomorphic (Fig. 1). APOV1 virions contain a major capsid protein of 10.5 kDa and two minor capsid proteins [3]. The droplet-shaped morphology is unprecedented among viruses of bacteria and eukaryotes and represents a group of archaea-specific virion morphotypes [4].

**Table 1.** Characteristics of the family *Guttaviridae*

Typical member:	Aeropyrum pernix ovoid virus 1 (HE580237), species *Aeropyrum pernix ovoid virus 1,* genus *Betaguttavirus*
Virion	Enveloped virions of ovoid shape, with a diameter of 55–80 nm and a length of 75–130 nm
Genome	Circular, dsDNA molecule of ~14–20 kbp
Replication	Genome is likely to be replicated by the host replisome
Translation	Not known
Host range	Hyperthermophilic archaea, phylum Crenarchaeota
Taxonomy	Two genera: *Alphaguttavirus* and *Betaguttavirus*

## Genome

**Fig. 2.**
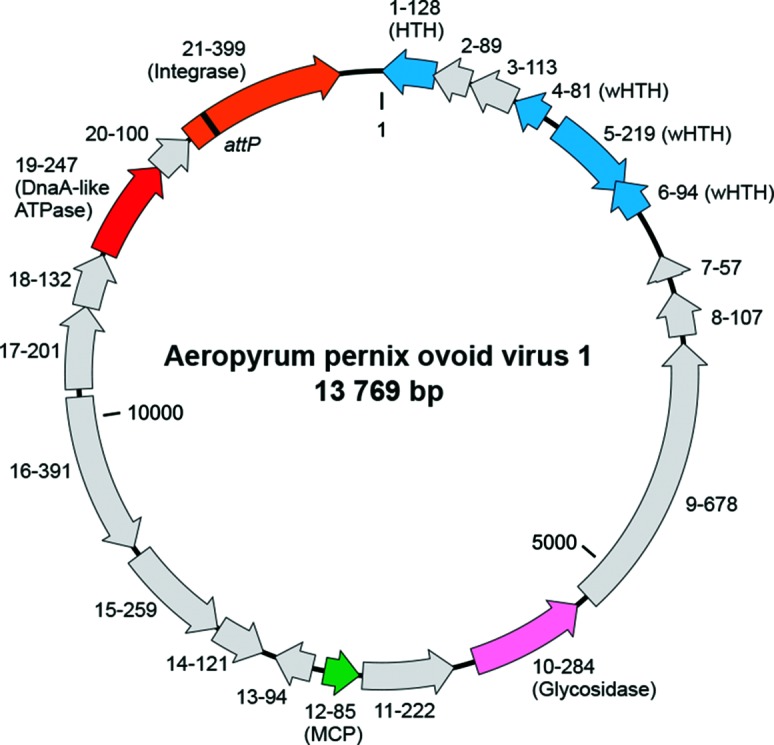
Genome map of Aeropyrum pernix ovoid virus 1. Functionally annotated ORFs are highlighted with different colours. Gene names include information on the length and function (when available) of the encoded proteins. Abbreviations: (w)HTH, (winged) helix-turn-helix DNA-binding proteins; MCP, major capsid protein.

The genome of guttaviruses consists of a circular dsDNA molecule. The SNDV genome is ~20 kbp and is known to be N(6)-methylated, but sequence information is not available [1]. The APOV1 genome is 13 769 bp, consistent with its smaller virion size compared to SNDV, and has a GC content of 56.5 % [2]. The genome contains 21 ORFs that could encode proteins of more than 56 amino acids, including an integrase of the tyrosine recombinase superfamily, a DnaA-like ATPase, a glycoside hydrolase and several DNA-binding proteins containing helix-turn-helix motifs (Fig. 2).

## Replication

Information on the replication cycle of guttaviruses is very scarce. APOV1 resides in the genome of *Aeropyrum pernix* as a provirus integrated into the *tRNA^Leu^* gene. Excision of the proviral APOV1 genome from the host chromosome, followed by genome replication and virion production, is induced under suboptimal growth conditions, namely, reduced aeration [2]. APOV1 does not carry a gene for a DNA polymerase, suggesting that its genome is replicated by the host replisome. Similarly, SNDV resides within the host cell in a carrier state as an episomal provirus, which is spontaneously induced at the early stationary growth phase. SNDV virion release is associated with host cell lysis [1].

## Taxonomy

APOV1 and SNDV infect hosts belonging to two different orders of the phylum Crenarchaeota. APOV1 was identified as a provirus integrated within the genome of *Aeropyrum pernix* strain K1 (order Desulfurococcales), which was isolated from a coastal solfataric vent at Kodakara-Jima Island, Japan. SNDV was discovered in a carrier state in a *Sulfolobus* strain (order Sulfolobales) isolated from a solataric field sample in Steaming Hill, New Zealand. The viruses are classified in the *Guttaviridae* family on the basis of the similar morphology and topology of their dsDNA genomes. However, the two viruses display distinguishable morphological features that justify their classification into two genera. A better understanding of *Guttaviridae* taxonomy will require the isolation of further family members. The bipartite gene-sharing network analysis of the archaeal virosphere showed that APOV1 forms a common module with fuselloviruses, indicating that the two groups of hyperthermophilic archaeal viruses might be evolutionarily related [5, 6].

## Resources

Full ICTV Online (10th) Report www.ictv.global/report/guttaviridae.
